# Wound Healing Fluid Reflects the Inflammatory Nature and Aggressiveness of Breast Tumors

**DOI:** 10.3390/cells8020181

**Published:** 2019-02-19

**Authors:** Roberto Agresti, Tiziana Triulzi, Marianna Sasso, Cristina Ghirelli, Piera Aiello, Ilona Rybinska, Manuela Campiglio, Lucia Sfondrini, Elda Tagliabue, Francesca Bianchi

**Affiliations:** 1 Division of Surgical Oncology, Breast Unit, Fondazione IRCCS Istituto Nazionale dei Tumori di Milano, 20133 Milan, Italy; Roberto.agresti@istitutotumori.mi.it; 2 Molecular Targeting Unit, Fondazione IRCCS Istituto Nazionale dei Tumori di Milano, 20133 Milan, Italy; Tiziana.triulzi@istitutotumori.mi.it (T.T.); Marianna.sasso@gmail.com (M.S.); ghirelli.cristina@istitutotumori.mi.it (C.G.); piera.chef@gmail.com (P.A.); ilona.rybinska@istitutotumori.mi.it (I.R.); manuela.campiglio@me.com (M.C.); francesca.bianchi@istitutotumori.mi.it (F.B.); 3 Dipartimento di Scienze Biomediche per la Salute, Università degli Studi di Milano, 20133 Milan, Italy; Lucia.sfondrini@unimi.it

**Keywords:** wound, extracellular matrix, cytokines, breast cancer, surgery, IL-6, G-CSF, osteopontin

## Abstract

Wound healing fluid that originates from breast surgery increases the aggressiveness of cancer cells that remain after the surgery. We determined the effects of the extent of surgery and tumor-driven remodeling of the surrounding microenvironment on the ability of wound-healing to promote breast cancer progression. In our analysis of a panel of 34 cytokines, chemokines, and growth factors in wound healing fluid, obtained from 27 breast carcinoma patients after surgery, the levels of several small molecules were associated with the extent of cellular damage that was induced by surgery. In addition, the composition of the resulting wound healing fluid was associated with molecular features of the removed tumor. Specifically, IP-10, IL-6, G-CSF, osteopontin, MIP-1a, MIP-1b, and MCP1-MCAF were higher in more aggressive tumors. Altogether, our findings indicate that the release of factors that are induced by removal of the primary tumor and subsequent wound healing is influenced by the extent of damage due to surgery and the reactive stroma that is derived from the continuously evolving network of interactions between neoplastic cells and the microenvironment, based on the molecular characteristics of breast carcinoma cells.

## 1. Introduction

Women with breast cancer undergo breast-conserving surgery or mastectomy as part of their treatment. The relationship between breast cancer surgery and the risk of relapse has been studied extensively. The risk of recurrence after primary breast cancer removal peaks at 10 months, implicating an event at the time of the surgery that accelerates or stimulates the metastatic process [1]. Several theories have been proposed to explain the mechanism that underlies early relapse on surgery. Metastatic processes that are triggered by breast tumor resection can not merely be ascribed to the release of tumor cells from the surgical bed [2]. The relevance of inflammation that is driven by wound healing is well established. Increasing evidence of a reduction in breast cancer relapses with the use of non-steroidal anti-inflammatory drugs (NSAIDs) has demonstrated the involvement of surgery-derived inflammation in metastatic processes [1,3,4]. 

One of the most obvious effects of wound healing is the stimulation of residual malignant cells or quiescent tumor stem cells by factors that are released in response to inflammation [5,6]. Moreover, angiogenic processes that are induced by the re-epithelization of wound tissue coincides with the generation of the vascular niche that supports tumor stem cell proliferation [7]. 

Following tissue injury via incision, the first step in the wound healing cascade is hemostasis, during which blood vessels constrict to limit blood flow, after which platelet aggregates and clotted blood impregnate the wound immediately to seal the lesion. During the inflammatory stage, an influx of immune cells ensues to control bleeding and prevent infection. Next, after the first 24 hours, angiogenesis and re-epithelization occur in the proliferation step to set down new cellular and extracellular matrix (ECM) components, in parallel with the release of cellular substances and mediators. Finally, the ECM is remodeled during the maturation phase [8]. 

Wound healing fluid (WHF) that results from surgical sites might provide a glimpse of the activity of cells that coordinate to release growth factors, cytokines, and chemokines that are fundamental in healing [9,10]. The composition of biological fluids in humans is influenced by their physiological characteristics, but several pathological conditions, such as metabolic disorders, can affect the levels of small molecules in these fluids [11]. 

During tumor formation and progression, cancer cells participate in dense crosstalk with all of the cell types that form the surrounding tissue and with the ECM that provides structural support [12]. The tumor stroma and ECM are considered essential for sustaining tumor growth because tumor cells foster changes to the surrounding niche through a complex network of signals, creating an environment that favors their proliferation and dissemination [13].

WHF can also force the escape of immune-controlled cancer cells from dormancy and promotes the transformation of already damaged cells [5,14,15,16]. Accordingly, we have established that growth factors that are released during wound healing following surgery account for the early relapse of the HER2-positive breast cancer subtype [17,18]. Moreover, we recently demonstrated that exposure of triple-negative (TN) breast cancer cells to WHF in vitro increases their expression of CDCP1, a molecule that is associated with a poor prognosis in several types of tumor, including TNBC [19,20].

At the time of tumor surgery, breast cancer cells have already modified the adjacent tissues extensively to create a microenvironment that favors tumor growth and dissemination. In this study, we examined whether the composition of WHF depends on and reflects remodeling of the ECM and the crosstalk with stromal cells provoked by tumor growth and whether such an interaction culminates in the release of molecules that are relevant to tumor aggressiveness into the wound fluid.

## 2. Materials and Methods

### 2.1. Collection of Drainage 

WHF from breast cancer patients who were treated surgically at Fondazione IRCCS Istituto Nazionale dei Tumori di Milano from 2010–2012 was collected from the first clearance of surgical closed-type breast drains (no abdomen or armpit) under suction during the first 24 h postsurgery. The WHF was centrifuged immediately at 3000 g, and the supernatant was aliquoted and stored at −80 °C until analysis. The protein concentration in the WHF, as determined by Biureto method, ranged from 3.7 to 5.1 g/dL. A human serum (HS) sample comprised a pool of HS from four healthy blood donors. The pathobiological features of the breast cancer patients, from whom WHF was collected, were registered, and a database was created.

The collection did not include WHF from patients with concomitant diseases other than breast cancer that are known to affect the release of small molecules into biological fluids (e.g., hyperglycemia) or patients who received chemotherapy or hormone therapy before surgery. 

### 2.2. Analysis of Small Molecules 

The composition of the WHF was analyzed on a Bio-Plex™ 2200 system (Bio-Rad Laboratories, Hercules, CA, USA), testing small-molecule signaling mediators, including cytokines, chemokines, and growth factors, that are involved in the initiation and progression of cancer. Specifically, the Bio-Plex Pro Human Cytokine 27-Plex Group I assay was used, including assays for PDGF-BB, IL-1b, IP-10, IL-1ra, IL-2, IL-4, IL-5, IL-6, IL-7, IL-8, IL-9, IL-10, IL-12p70, IL-13, IL-15, IL-17, eotaxin, FGF basic, G-CSF, GM-CSF, IFN-g, MCP-1MCAF, MIP-1a, MIP-1b, RANTES, TNF-α, and VEGF. In the indicated experiments, this panel of analytes was integrated with assays for PDGF-AB/BB, sTIE-2, HGF, osteopontin, TGF-beta1, TGF-beta2, and TGF-beta3. The analysis was performed according to the manufacturer’s instructions and as described [21,22,23]. 

Briefly, samples were incubated in a 96-well plate with polystyrene beads that were coated with small molecule-specific antibodies and then exposed to detection antibodies prior to incubation with streptavidin-PE. Data are presented as concentration (pg/mL). The concentration of each analyte, bound to its specific bead, is proportional to the median fluorescence intensity (MFI) of the reporter signal. All samples were assayed in duplicate.

### 2.3. Cell Lines, Cultures, and Treatments

The human breast cancer cell lines were maintained at 37 °C in a humidified atmosphere of 5% CO_2_ in air as follows: MDA-MB-231, BT-549, SK-BR-3, HCC1937, T-47D and MCF-7 in RPMI 1640 (Life Technologies, Grand Island, NY, USA) and MDA-MB-468 in Dulbecco’s modified Eagle’s medium (DMEM) (Lonza, Basel, Switzerland). For the stimulation with WHF, cells were starved in serum-free medium for 24 h and then treated with WHF that was diluted in culture medium and passed through a 0.22-µm syringe PVDF filter (Millipore, Burlington, MA, USA) [19,20].

### 2.4. In Vitro Growth and Migration Assays 

Relative 2D cell growth over 4 days was measured by sulforhodamine B (SRB) assay as described [24]. Optical density (OD) was determined on an ELISA microplate reader (Bio-Rad Laboratories). Proliferation was spectrophotometrically assessed by SRB assay after 4 days of treatment (96 h). For each cell line, the optical density (OD) of each experimental condition was normalized on the OD of the same cell line measured immediately before starting the treatments. 0% represents the growth index of cells cultured for 4 days in absence of WHF or fetal bovine serum (FBS) or HS.

To examine the ability of WHF to enhance cell migration, cells were starved in serum-free medium for 48 h, treated with or without a 5% WHF pool for 2 h, and then seeded at the top of a 8-µm Boyden chamber (Sigma-Aldrich) in serum-free medium, with medium that contained 10% FBS placed in the well below as the chemoattractant. After 12 h for MDA-MB-231 cells or 6 h for BT-549 cells, the cells in the upper chamber were removed with cotton swabs, and those that traversed the 8-µm semipermeable membrane were fixed in 100% ethanol, stained with SRB, and imaged under an ECLIPSE TE2000-S inverted microscope (Nikon Instruments, Amstelveen, Netherlands). The results were expressed as the area that was occupied by cells in the bottom of the Transwell, evaluated by digital image analysis using the appropriate software (Image Pro-Plus 7.0 application, Media Cybernetics, Rockville, MD, USA). The mean of 3 independent experiments (± SEM) was calculated.

### 2.5. Statistical Analysis

Relationships between categorical variables in Table S1 that were related to primary breast cancers from which the WHF was derived were analyzed by Fisher’s exact test. To compare the levels of small molecules between 2 independent groups, mean values were compared by nonparametric Mann–Whitney test. The effect of WHF on cell migration was analyzed by student’s *t*-test. All analyses were performed using GraphPad Prism, version 5.0 for Windows (GraphPad Software, San Diego, CA, USA). Differences were considered to be significant at *p* ≤ 0.05. 

Overall survival (OS) was defined as the time elapsed from the date of surgery to the date of death. Distant metastasis free survival (DMFS) was defined as the time elapsed from the date of surgery to the date of the first event. Univariate survival analysis was carried out using Cox proportional hazards regression models, and the effects of explanatory variables on event hazard were quantified by hazard ratios (HR). Small-molecules amount was analyzed after logarithmic transformation (log2). Analysis has been performed by using SAS software (SAS Institute Inc, Cary, NC, USA).

## 3. Results

### 3.1. Analysis of the Composition of WHF in Breast Cancer Patients

**Figure 1:**
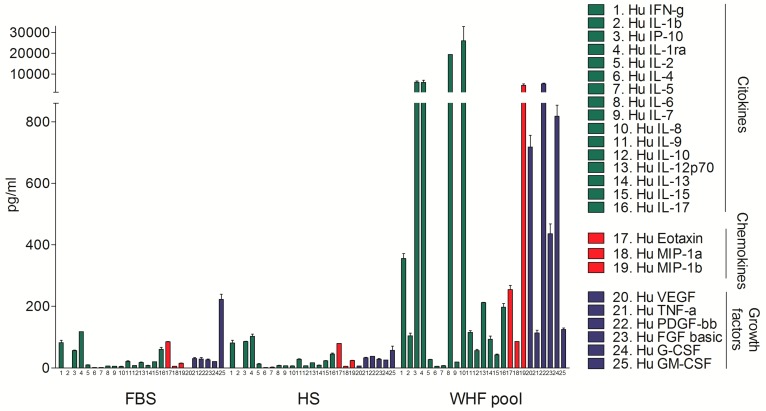
Bio-plex analysis of fetal bovine serum, human serum, and wound healing fluid composition. Concentration (pg/mL) of 25 molecules is shown. Level of cytokines, chemokines, and growth factors, was assessed by Bio-plex assay in fetal bovine serum (FBS), human serum (HS), and a pool of five wound healing fluids from breast cancer surgery (WHF Pool) (mean ± SD).

To determine the feasibility of analyzing the small molecules in WHF at the site of breast cancer surgery, a pool of 5 breast cancer WHF samples, a pool of 4 human serum (HS) samples, and fetal bovine serum (FBS) were evaluated by a Bio-Plex Pro Human Cytokine 27-Plex Group I assay. The WHF pool was enriched in cytokines, chemokines, and growth factors compared with FBS and HS. Overall, the peak concentration of all small molecules in the HS was approximately 100 pg/mL, as expected, based on the literature, whereas that of over half of the analyzed molecules in WHF (17/25) was higher than 100 pg/mL, six of which—MIP-1b, PDGF-bb, IL-1ra, IP-10, IL-6, and IL-8—exceeded 1000 pg/mL. HS and FBS did not differ significantly with regard to any molecule (Figure 1).

**Figure 2:**
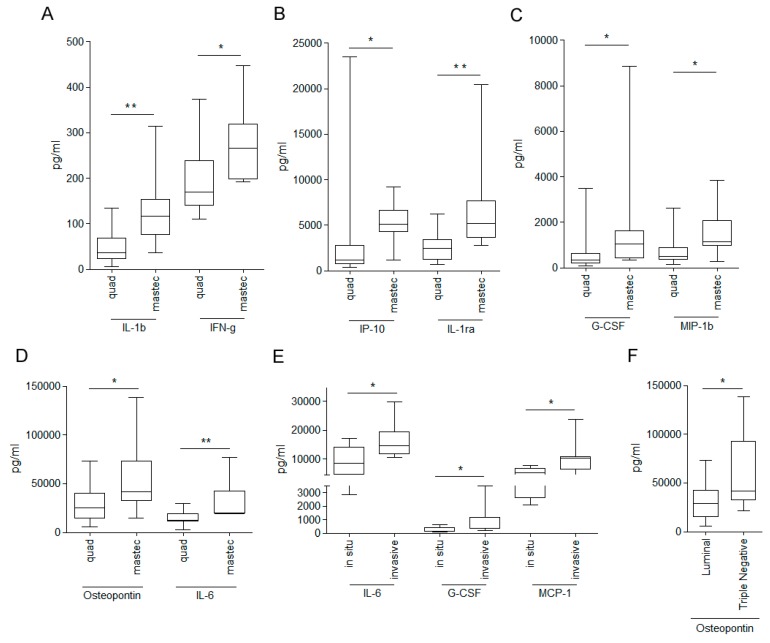
Differences in small-molecule composition of wound healing fluid from breast carcinoma surgery according to type of surgery, tumor histology, and tumor molecular subtype. Concentration (pg/mL) of 34 molecules, including cytokines, chemokines, and growth factors (listed in Material and Methods), was assessed by Bio-plex assay in 27 wound healing fluids from breast cancer patients. Levels of small molecules were differentially enriched by surgery (**A**–**D**), tumor histology (**E**), and tumor molecular subtype (**F**) (* *p*-value ≤ 0.05; ** *p*-value ≤ 0.01; Mann–Whitney test).

To dissect the composition of fluids that are released immediately after breast cancer surgery, the levels of 34 small molecules were analyzed in a collection of 27 WHF samples (Table S1) by Bio-Plex Pro Human Cytokine 27-Plex Group I assay, integrated with a panel of nine additional small molecules (see Materials and Methods). Several small molecules were differentially expressed in WHF from breast cancer patients who underwent mastectomy or quadrantectomy. Specifically, the levels of IL-1b, IL-1ra, IL-6, osteopontin, IFN-γ, G-CSF, MIP-1b, and IP-10 were significantly higher in mastectomized patients than in those who underwent quadrantectomy (Figure 2A–D). Frequency analysis showed that the type of surgery was not significantly associated with any pathological variable, despite a near-significant trend for a negative association between mastectomy and the luminal tumor intrinsic molecular subtype and for a positive association between mastectomy and invasive tumor (Table S2). 

Considering that all patients with in situ breast cancer in our case study underwent breast-conserving surgery, the composition of WHF regarding tumor histology was analyzed according to quadrantectomy. In patients who underwent quadrantectomy, IL-6, G-CSF, and MCP1-MCAF were significantly enriched in invasive versus in situ breast cancer surgery drainages (Figure 2E), suggesting that the crosstalk between invasive tumor cells and the surrounding microenvironment influences the production and release of these small molecules.

With regard to intrinsic subtype tumors, osteopontin (OPN) was significantly higher in fluids from TN (defined as estrogen receptor (ER) and/or progesterone receptor (PgR) <10% and HER2 0, 1+, 2+ CISH-negative) compared with luminal breast cancer (defined as ER and PgR expression >10% and HER2 0, 1+, 2+ CISH-negative) (Figure 2F). Notably, albeit insignificantly, the level of OPN was higher in fluids from TN breast cancer patients, independent of the type of breast tumor surgery (Figure S1).

**Figure 3:**
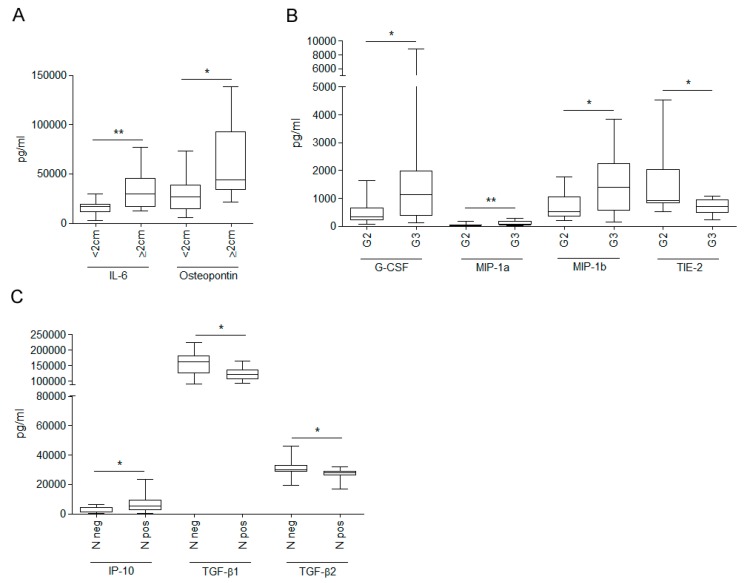
Differences in small-molecule composition of wound healing fluid from breast carcinoma surgery according to tumor size, tumor grade, and lymph node positivity. Concentration (pg/mL) of 34 molecules, including cytokines, chemokines, and growth factors (listed in Material and Methods), was assessed by Bio-plex assay in 27 wound healing fluids from breast cancer patients. Levels of small molecules were differentially enriched by breast cancer size (**A**), tumor grade (grade II (G2); grade III (G3) (**B**), and lymph node (**C**) (* *p*-value ≤ 0.05; ** *p*-value ≤ 0.01; Mann–Whitney test).

IL-6 and OPN were significantly enriched in WHF from surgeries for breast cancers ≥ 2 cm (Figure 3A). G-CSF, MIP-1a and MIP-1b content was significantly higher and TIE-2 was significantly lower in grade III versus grade II breast tumors (Figure 3B). Level of IP-10 was significantly upregulated, whereas TGF-β1 and TGF-β2 were significantly lower, in WHF from N-positive compared with N-negative tumors (Figure 3C).

In our cohort, none of the clinical characteristics of breast cancer patients, from which wound healing fluids have been derived, were associated with prognosis. Nevertheless, a trend towards significance was found for association of IP-10 and OPN amount with both DMFS (IP-10, HR: 1.97, 95% Confident Interval (CI): 0.86–4.55, *p* = 0.1104; OPN, HR: 2.67, 95% CI: 0.98–7.25, *p* = 0.0537) and OS (IP-10, HR: 2.15, 95% CI: 0.86–5.34, *p* = 0.1006; OPN, HR: 2.95, 95% CI: 1.06–8.22, *p* = 0.0381). 

### 3.2. Relationship between WHF and Cancer Cell Aggressiveness

**Figure 4:**
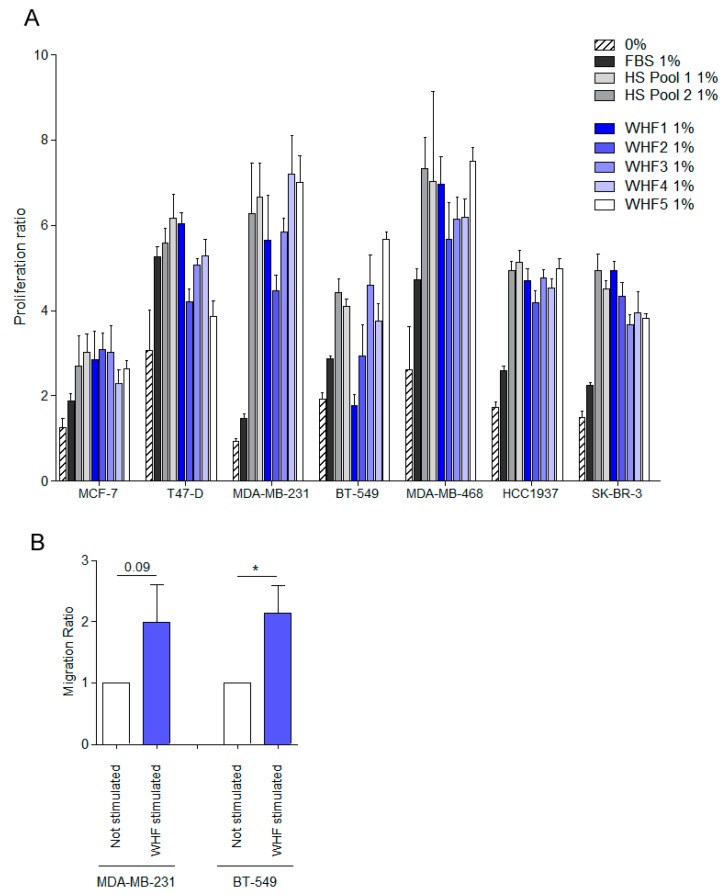
Effect of WHF on breast cancer cell proliferation and migration. (**A**) MCF-7, T-47D, MDA-MB-231, BT-549, MDA-MB-468, HCC1937, and SK-BR-3 breast cancer cell lines were starved for 24 h (0% FBS) and then treated for 96 h with 1% FBS or 1% of pools of human serum from breast cancer patients or 1% of five WHFs. Relative 2D-cell growth was measured by sulforhodamine B (SRB) assay. The ability of WHF to induce proliferation was indicated as the optical density (OD) of each cell line after four days of treatment (96 h) normalized on the OD of the same cell line measured immediately before starting the treatments. 0% represents the growth index of cells cultured for four days in absence of WHF or FBS or serum from breast cancer patients (HS Pool) (mean ± SD). (**B**) MDA-MB-231 and BT-549 cells were starved for 48 h (0% FBS) and then treated for 2 h with a pool of five WHFs and plated into Boyden chambers for migration assay toward FBS 10%. The area occupied by migrated cells in the Transwell was evaluated by digital image analysis (Image ProPlus 7.0 application, Media Cybernetics). Results are expressed as area occupied by cells on the bottom of the Transwell by digital image analysis (mean ± SEM; * *p*-value < 0.05; paired student’s *t*-test).

To determine the effects of WHF on cell growth, a panel of seven breast cancer cell lines, representing various breast cancer subtypes—MDA-MB-231 and BT-549 (basal-B TN subtype), MDA-MB-468 and HCC1937 (basal-A TN subtype), SK-BR-3 (HER2 subtype), MCF-7, and T-47D (luminal subtype) [25]—were starved for 24 h, treated with 1% FBS or 1% of pools of human serum from breast cancer patients or of five different WHFs for 96 h, or left untreated. The WHF stimulated robust cell growth in all cell lines (Figure 4A). Similar results were obtained using a wide panel of 45 WHFs tested on four breast cancer cells—MDA-MB-468, MDA-MB-231, BT-549 and MCF-7—strengthening the effect of WHF on cell proliferation (Figure S2).

Because several chemokines were enriched in WHF, its ability to promote migration was also examined in the basal-B TN breast cancer cell lines MDA-MB-231 and BT-549, which have been reported to migrate in vitro. Briefly, cells were starved for 48 h, treated with pools of WHFs for 2 h, or left untreated and seeded in a chamber assay that contained FBS in the lower chamber as the chemoattractant (Figure 4B). In both cell lines, migration was improved on pretreatment with WHF.

### 3.3. Relevance of Breast Cancer Subtype in WHF-Driven Stimulation

**Figure 5:**
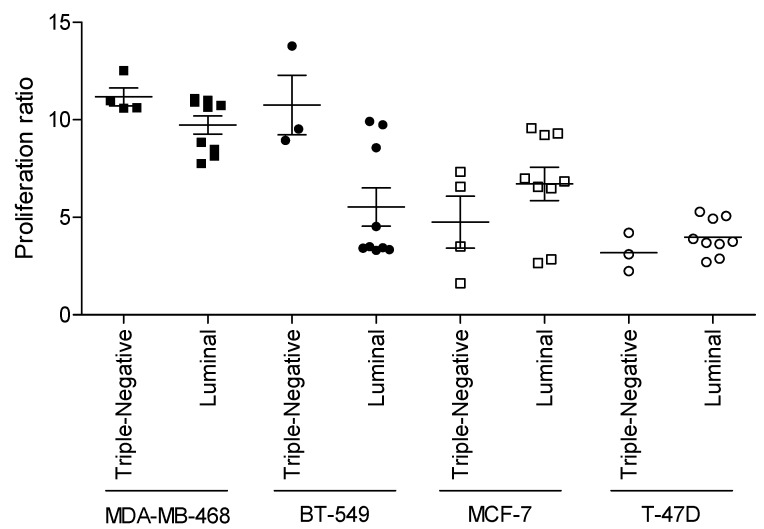
Effect of WHF originating from triple-negative or luminal breast cancer surgery on proliferation of triple-negative- or luminal-subtype breast cancer cells. The TN breast cancer cell lines MDA-MB-468 and BT-549 and the luminal breast cancer cell lines MCF-7 and T-47D were starved for 24 h (0% FBS) and then treated for 96 h with 1% WHFs from patients bearing TN or luminal breast cancer. WHF-induced cell growth was analyzed according to resected breast cancer subtype. The ability of WHF to induce proliferation was indicated as the ratio of the OD of each cell line (WHF-treatedat 96 h/OD at 0%, before treatment).

To determine whether the WHF from breast tumor surgery for various molecular subtypes of tumors preferentially affects the proliferation of cancer cells with the same molecular characteristics, the TN luminal breast cancer cell lines MDA-MB-468 and MCF-7 were stimulated as described with WHF from TN or luminal breast cancer surgery, and proliferation was evaluated by SRB assay (Figure 5). In addition to the difference in intrinsic proliferation rate between the cell lines—the proliferation rate of MDA-MB-468 cells was higher than that of T-47D cells, as expected—the proliferation of MDA-MB-468 and BT-549 cells increased primarily by WHF from the surgical removal of TN versus luminal breast tumors. Similarly, the proliferation of MCF-7 and T-47D that was induced by luminal-derived WHF was greater than that by TN breast tumors. These data support our hypothesis that tumors per se can modify their environment to favor their progression.

## 4. Discussion 

We have demonstrated that surgery-induced mobilization of factors is related to the biological features of the breast tumor that is removed. The remodeling of the microenvironment by tumor cells is reflected in the small-molecule composition of WHF. Several protumorigenic small molecules were upregulated in WHF from breast cancers with aggressive features versus less aggressive tumors; such molecules can ultimately cooperate to establish conditions that favor relapse. As a support, a higher amount of molecules associated with tumor aggressive features was found in patients with worse event free survival and overall survival.

Recently, the effects of wound healing were recently found to depend primarily on the subsequent inflammatory response, independent of the nature of the resected tumor [16]. By testing surgical wounds regardless of the presence of tumor, wound healing has been shown to activate distant disseminated tumor cells. Clearly, the transient acute inflammation that is provoked by surgery is a systemic event, and factors that are released at the surgical site, such as cytokines and chemokines, can reach, through the circulatory system, and stimulate cancer cells that have already disseminated at the time of the primary tumor resection. 

Nevertheless, we highlight the function of the crosstalk between the tumor and its microenvironment, as we previously observed in other tumor types [26,27]. Several cell types, including fibroblasts, endothelial cells, and immune cells, interact with tumor cells, and their activity in shaping the tumor microenvironment is reflected in the composition of the wound healing fluid that is collected during the first 24 h after breast cancer surgery. In our cohort, differences in WHF composition were observed with regard to primary breast tumor histology, intrinsic subtype, size, grade, and lymph node status. Particularly, small molecules with reported functions in tumor inflammation and progression were enriched in WHF from the surgery of tumors with more aggressive features. 

In this context, IL-6, G-CSF, and MCP-1 were enriched in WHF from patients with invasive versus in situ breast cancer, independent of the extent of the surgery. Consistent with its function in cancer progression, IL-6 was also upregulated in WHF from larger late-stage tumors. IL-6 regulates the tumor microenvironment, the generation of breast cancer stem cells, and metastasis through the downregulation of E-cadherin [28]. Increased serum IL-6 was correlated with survival [29]. 

Notably, G-CSF was enriched in WHF from invasive and high-grade breast tumors. G-CSF is one of the chief growth factors that control the maturation of neutrophils. Elevated neutrophil levels have been associated with detrimental outcomes in breast cancer, such that neutrophil count has been proposed as a prognostic and predictive biomarker [30]. Neoplastic cells draw in neutrophils that are recruited to a wound, increasing their proliferation [31]. 

IP-10/CXCL10, enriched in N-positive tumors, has several functions, such as chemoattraction for immune cells and the promotion of angiogenesis [32]. Notably, the levels of TGF-β1 and TGF-β2, reportedly acting as potent growth inhibitors [33], were reduced in N-positive tumors. With regard to the composition of WHF from the surgery bed following the removal of various molecular subtypes of tumor, the matricellular protein osteopontin (OPN, Spp-1) was the only small molecule that differed in concentration between WHFs from luminal and TN breast cancer. Nevertheless, the small size of WHF cohort, a trend towards significance was found for association of IP-10 and OPN amount with both event free survival and overall survival, supporting the hypothesis of the role of these molecules in tumor aggressiveness. OPN acts as an immune modulator, promoting neutrophil and mast cell recruitment to inflammatory sites. In addition, OPN mediates cell activation and cytokine production and enhances cell survival by regulating apoptosis [34,35]. OPN is overexpressed in breast cancer, particularly the TN subtype [36,37]. Recently, it was discovered that OPN is the basis for one of the mechanisms of breast cancer cell metastasis. OPN that is produced by tumor cells supports their survival in the bloodstream, whereas tumor- and host-derived OPN, particularly from myeloid cells, render the metastatic site more immunosuppressive [38]. The enrichment of OPN in TN tumor fluids could reflect its roles in tumor survival and spreading promotion and explain the high recurrence rate of this breast cancer subtype. 

WHF stimulates in vitro proliferation of breast cancer cells of all intrinsic subtypes, consistent with what was reported by Wang and colleagues [39]. Although the increase in proliferation by WHFs was similar between cancer cell lines of various subtypes, the effect of proliferation was greater when the cells were treated with WHF from the surgery of tumors of the same intrinsic subtype. This effect was observed in more aggressive TN cell-WHF pairs and in less proliferating luminal tumors. These data suggest that breast tumors can purposely condition the extracellular microenvironment and, consequently, the factors in WHF. 

We compared the levels of small molecules found in our WHF with data reported in literature. D.E. Lyon and colleagues [40], by using the same Multiplex bead array assay we used, analyzed levels of 17 cytokines in serum of women with breast cancer and of women with a suspicious breast mass who were found subsequently to have a negative breast biopsy. They observed a significantly higher cytokine concentration in women with cancer compared to women without cancer for the majority of the analyzed cytokines. Interestingly, comparing the cytokine levels of our WHF with those found in serum by Lyon et al., five cytokines (IL-6; IL-8; G-CSF; IFN-γ; MCP-1) were highly enriched in WHF compared with their serum counterpart (data not shown). Here, we found that these five cytokines were found differently represented in WHFs according to type of surgery, tumor histology, and tumor molecular subtype. Altogether, we can speculate that specific WHF cytokines that are related to tumor aggressiveness are also enriched in serum of BC patients. Consistently, we observed that pools of breast cancer sera increase proliferation of breast cancer cells similarly to what was observed upon WHF treatment. Factors released in the tumor microenvironment, as a consequence of a tumor’s own growth and of interaction with stroma and with surrounding cells (immune cells, fibroblasts, etc.), enter the bloodstream and could contribute to promoting tumor growth and dissemination. Moreover, we can assume that the cytokines we found more represented in WHF compared to serum levels reported in the literature presumably act on tumor aggressiveness features other than proliferation, such as immune tumor control. 

Finally, our data show that the composition of fluid that is released immediately after the surgery of breast cancer patients mirrors the extent of surgery, and, in particular, all small mediators that we have described were enriched in WHF from patients who underwent mastectomy. Surgery is a key cancer therapy and remains the most effective treatment for breast cancers. The two main types of surgery to remove breast cancer are quadrantectomy and mastectomy. A minimal surgical approach, where applicable, will benefit patients by reducing stimulation of inflammation. Several inflammatory mediators were enriched in the drainage of patients who underwent mastectomy, supporting that a highly destructive surgery increases inflammation [41,42]. Moreover, more reports are indicating that tissue damage due to cancer surgery provides a favorable niche for tumor recurrence [43], facilitating the growth of pre-existing micrometastases [5,18,44,45], enhancing the cancer stem cell population [6], creating a reactive oxygen species (ROS)-rich environment [46], and affecting patient outcomes.

## 5. Conclusions

Our data, in addition to confirming previous evidence that inflammation that stems from surgical wound healing affects breast cancer cell aggressiveness, highlight the relevance of tumor-induced modifications to the surgical bed. Breast cancers with aggressive features can specifically modify the tumor environment to ultimately favor their growth. Because surgery remains the preferred option for treating cancer, it is essential to improving our understanding of the inflammatory response that occurs as a consequence of local wounding and of the exposure of cancer cells to wounds. 

## Supplementary Materials

The following are available online at https://www.mdpi.com/2073-4409/8/2/181/s1, Table S1: Supplementary Table 1. Clinical characteristics of breast cancer patients from which wound healing fluids were derived. Table S2: Supplementary Table 2. Clinical characteristics of breast cancer patients from which wound healing fluids were derived according to extent of surgery. Figure S1: Supplementary Figure 1. Osteopontin levels in wound healing fluid from breast carcinoma patients according to breast tumor molecular subtype and type of surgery. Figure S2: Supplementary Figure 2. Effect of WHF on breast cancer cell proliferation.
