# Supplementary material for: Wound Healing Fluid Reflects the Inflammatory Nature and Aggressiveness of Breast Tumors
Source: Cells. 2019 Feb 19;8(2):181. doi: 10.3390/cells8020181 (PMC6406730; doi:10.3390/cells8020181)
Supplement: Supplementary file 1 [file cells-08-00181-s001.pdf]

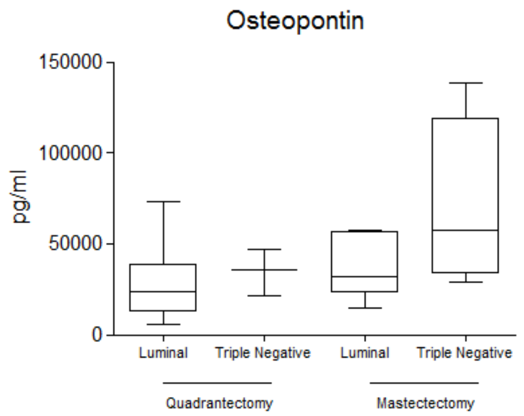

Supplementary Figure 1. Osteopontin levels in wound healing fluids from breast carcinoma patients according with breast tumor molecular subtype and type of surgery. Concentration (pg/ml) of Osteopontin was assessed by Bio-plex assay in 27 wound healing fluids from breast cancer patients.

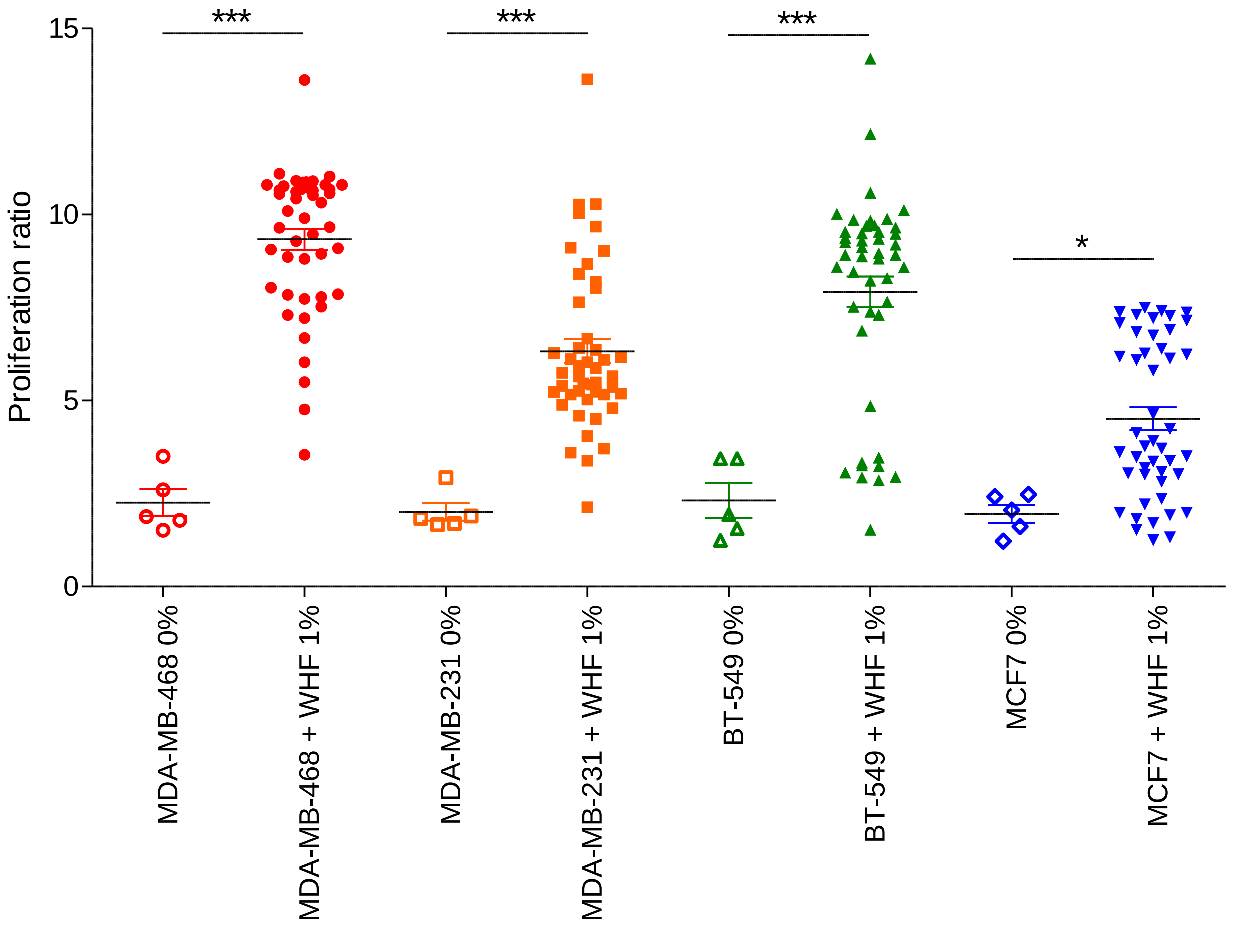

Supplementary Figure 2. Effect of WHF on breast cancer cell proliferation. MDA-MB-468, MDA-MB-231, BT-549 and MCF-7 breast cancer cell lines were starved for 24 h (0% FBS) and then treated for 96 h with 1% of 45 WHFs. Data about chemotherapy or hormone therapy before surgery were not known. Relative 2D-cell growth was measured by sulforhodamine B (SRB) assay. Ability of WHF to induce proliferation was indicated as the OD of each cell line after 4 days of treatment (96 h) divided by the optical density before WHF treatment. 0% represents the growth index of cells cultured for 4 days in absence of WHF (n=5). (* p value ≤0.05; *** p value < 0.0001; Unpaired t test).

Supplementary Table 1. Clinical characteristics of breast cancer patients from which wound healing fluids have been derived.

	Overall cohort (N= 27)
Surgery	
Quadrantectomy	16 (59%)
Mastectomy	11 (41%)
Histology	
In situ	5 (19%)
Invasive	22 (81%)
Intrinsic subtype	
Luminal	18 (67%)
Triple-Negative	9 (33%)
Size	
<2 cm	17 (63%)
≥2 cm	9 (33%)
na	1
Grade	
2	15 (56%)
3	12 (44%)
Lymph node	
N neg	16 (59%)
N pos	11 (41%)

Supplementary Table 2. Clinical characteristics of breast cancer patients from which wound healing fluids have been derived according with the extend of surgery.

	Overall cohort (N= 27)	Quadrantectomy (N= 16)	Mastectomy (N= 11)	P value[a]
Histology				
In situ	5 (19%)	5 (31%)	0 (0%)	0.0598
Invasive	22 (81%)	11 69%)	11 (100%)	
Intrinsic subtype				
Luminal	18 (67%)	13 (81%)	5 (45%)	
Triple-Negative		3 (19%)	6 (55%)	0.0969
	9 (33%)			
Size				
<2 cm	17 (63%)	11 (69%)	6 (55%)	0.4185
≥2 cm	9 (33%)	4 (25%)	5 (45%)	
na	1	1	-	
Grade				
2	15 (56%)	10 (62%)	5 (45%)	0.4517
3	12 (44%)	6 (38%)	6 (55%)	
Lymph node				
N neg	16 (59%)	11 (69%)	5 (45%)	0.2638
N pos	11 (41%)	5 (31%)	6 (55%)	
